# The Evolution of the Multicoloured Face of Mandrills: Insights from the Perceptual Space of Colour Vision

**DOI:** 10.1371/journal.pone.0029117

**Published:** 2011-12-21

**Authors:** Julien P. Renoult, H. Martin Schaefer, Bettina Sallé, Marie J. E. Charpentier

**Affiliations:** 1 Department of Evolutionary Biology and Animal Ecology, Faculty of Biology, University of Freiburg, Freiburg, Germany; 2 Centre de Primatologie, Centre International de Recherches Médicales, Franceville, Gabon; 3 Centre d'Ecologie Fonctionnelle et Evolutive UMR 5175, Centre National de la Recherche Scientifique, Montpellier, France; University of Sussex, United Kingdom

## Abstract

Multicomponent signals consist of several traits that are perceived as a whole. Although many animals rely on multicomponent signals to communicate, the selective pressures shaping these signals are still poorly understood. Previous work has mainly investigated the evolution of multicomponent signals by studying each trait individually, which may not accurately reflect the selective pressures exerted by the holistic perception of signal receivers. Here, we study the design of the multicoloured face of an Old World primate, the mandrill (*Mandrillus sphinx*), in relation to two aspects of signalling that are expected to be selected by receivers: conspicuousness and information. Using reflectance data on the blue and red colours of the faces of 34 males and a new method of hue vectorisation in a perceptual space of colour vision, we show that the blue hue maximises contrasts to both the red hue and the foliage background colouration, thereby increasing the conspicuousness of the whole display. We further show that although blue saturation, red saturation and the contrast between blue and red colours are all correlated with dominance, dominance is most accurately indicated by the blue-red contrast. Taken together our results suggest that the evolution of blue and red facial colours in male mandrills are not independent and are likely driven by the holistic perception of conspecifics. In this view, we propose that the multicoloured face of mandrills acts as a multicomponent signal. Last, we show that information accuracy increases with the conspicuousness of the whole display, indicating that both aspects of signalling can evolve in concert.

## Introduction

It is becoming increasingly clear that animals frequently communicate by complex displays [1]–[3]. Complex displays can consist of different signals that are perceived individually, each signal conveying its own information. There is growing evidence, however, that complex displays can be perceived as a whole [4]–[8]. Such displays are often termed multicomponent signals because information conveyed by the whole display may be different or more accurate than information associated to the individual traits (named components hereafter) that make up the display [9]. The evolution of components in a multicomponent signal is expected to be mutually dependent and driven by the holistic perception of the display by receivers. The evolution of multicomponent signals has been mostly studied considering each component individually, so far [3]. By contrast, little is known on how the holistic perception of receivers effectively shapes the design of multicomponent signals.

Recently a few studies have investigated the inter-dependent evolution of signal components in complex displays in birds [10]–[12]. These studies used perceptual spaces of colour vision, which are geometrical representations of how receivers perceive colours [13]. Results are consistent with that the colours of body patches that are displayed to conspecifics have evolved to increase the overall conspicuousness of the whole display. Accordingly, individual plumage colours can thus qualify as signal components and multiple plumage colours as multicomponent signals. These studies further provided a framework to predict the evolution of signal components: they propose that new components that are added to a colour display over time should be selected to contrast against pre-existing colours, thereby maximizing the conspicuousness of the whole display.

A limitation of these studies is that the design of signals is not selected by receivers based on conspicuousness only but also to convey accurate information about signaller [3], [14]–[16]. In cuckoos, for example, chicks vary simultaneously gape size and calling rate depending on their food needs. Integration of these two traits by parents allows a more accurate assessment of food needs than could be determined from either trait alone [4]. This conjecture termed ‘increased information accuracy’ hypothesis hereafter predicts that perceiving a complex display as a whole allows a better assessment of information conveyed by this display.

In general, selection to increase information accuracy can interfere with selection to increase conspicuousness [14]. Doucet et al. [17] suggested that in manakins, the most conspicuous patches are also the least informative because of a trade-off between conspicuousness and accuracy, although these authors did not propose a mechanism for this trade-off. Empirical studies are needed to determine whether and how selection to increase the accuracy of signal information is combined with selection to increase conspicuousness in shaping multicomponent signals.

The goal of this study is to investigate whether selection to increase conspicuousness and/or information accuracy has shaped the multicoloured face of mandrills (*Mandrillus sphinx*). Male mandrills are unique among mammals in displaying an amazingly blue and red coloured face. As quoted by Darwin, “no other member in the whole class of mammals is coloured in so extraordinary a manner as the male mandrill” [18]. The information contained in the red colouration in primates has been extensively studied (e.g., [19], [20]). In male mandrills, the intensity in red colouration is positively correlated with both dominance [21] and female preference [22]. In contrast, the conspicuousness of red and blue patches and the information associated with blue colouration are unknown, even though blue colouration has been reported to vary between males [23]. We hypothesise that both red and blue patches convey information and, because they are adjacent on the face, that together these two colours form a multicomponent signal.

In this article, we first present a new and simple method based on the vectorisation of hues in a perceptual space of colour vision to predict colours that maximise the conspicuousness of the overall display given one or several pre-existing colours. Second, we analyse at the population level the correlations between dominance rank and colour attributes (i.e. hue and saturation) of the red and blue patches. Finally, we investigate whether and how the holistic perception of the multicoloured face of male mandrills can increase the accuracy of the perceived information.

## Materials and Methods

### Ethics Statement

We studied three semi-free ranging groups of mandrills housed at the ‘Centre International de Recherches Médicales de Franceville’, Gabon (CIRMF) in naturally forested enclosures of 6.5- (E1), 3.5- (E2) and 0.5-ha (E3). Mandrills live in three semi-free ranging social groups that are housed enclosures of 6.5, 3.5 and 0.5 ha. The densities are 0.0013, 0.0022, 0.04 ind/m^2^, respectively, which meet the recommendations of the Weatherall report for the use of non-human primates in research (0.5 ind/m^2^ for macaques). Enclosures are naturally enriched with rainforest. The care staff includes 15 assistants and 4 vets for a total of 400 non-human primates. Animals forage freely and are daily supplemented with fruits and monkey chow. All individuals are annually captured for routine physicals to tag newborns, for general medical examination and to check the viral status to SIV/STLV (natural transmission). The collection of data used in this study was performed during such a physical examination, was not invasive and did not entail any extension of the anesthesia initially planned by the veterinary team.

### Study population

The history of the breeding colony is detailed in Charpentier *et al.* [24]. In March 2010, the first (N = 85), second (N = 80) and third (N = 20) group comprised 9, 26 and 4 sexually mature males, respectively. Males were treated as sexually mature if they belonged at least to the birth-cohort born 6 yrs ago following Setchell and Dixson [25].

### Colour face measurements

In March 2010, we recorded colour spectra of 34 mature males (9, 24 and 1 from the first, second and third group, respectively) aged 5.9–21 yrs. Males were anaesthetized by blowpipe intramuscular injections of ketamine (Imalgène® 1000; 10 mg/kg) during a routine physical examination. Ketamine has a direct vasodilatative effect but also causes sympathetically mediated vasoconstriction. The net effect is that systemic vascular resistance is not significantly affected [26]. It is therefore unlikely that the red vasculary mediated colouration is significantly altered by Ketamin. We used an Ocean Optic USB2000 spectrometer and a Top Sensor Systems deuterium-halogen DH-2000 as a standardized light source. Reflectance measures were proportional to a white tile (WS-S2) standard. We measured facial reflectance spectra with a coaxial fibre cable mounted inside a matt black plastic tube. Recordings were made at an angle of 90° since neither red nor blue colours are glossy [27]. For each individual, we averaged the blue reflectance spectra and the red reflectance spectra from measures made at four different locations on each facial patch.

### Reflectance spectra analyses

We analysed reflectance spectra in the a*b* dimension of the CIELAB perceptual space developed for human colour vision [28]. Variation along a* and b* describes redness/greenness and blueness/yellowness variations, respectively. Hue is defined by an angle *H* = arctan(b*/a*). Colour saturation is defined as *C* = (a* ^2^+b* ^2^)^1/2^. The chromatic contrast between two colours (*CC*) is defined as the Euclidean distance between them in the space. We used a CIELAB perceptual space with a CIE 2° observer and a neutral illuminant, the “canopy cloudy” of Regan *et al.* [29]. In the CIELAB space, hue and saturation are scaled so that anywhere in the space, one unit of any combination of these attributes represents an approximately equal and just noticeable difference (JND) in colour perception [30]. In the discussion, we will argue that the CIELAB space is particularly suited for the analyses performed in this study. However, CIELAB has been designed for humans, and it is therefore not clear whether it is suited for other trichromatic primates like mandrills. We therefore performed the analyses again using another model of perceptual space, the Receptor-Noise Limited (RNL) model [31], which can include mandrill-specific data on the peripheral visual system. Results obtained with the RNL model are presented in Text S1 and are qualitatively similar to those obtained with the CIELAB model.

### Signal conspicuousness

The overall conspicuousness of a colour pattern is indicated in a perceptual space of colour vision by the distance among the distinct colours that form the pattern (e.g., see [10], [12], [17]). An important factor determining this distance is the hue disparity [12], which represents the diversity of colours as indicated by their vernacular meanings, like yellow, green or blue. Hues that maximally contrast with each other are opposite in the CIELAB space, i.e. they differ by 180° [30]. For each individual we plotted the average red and blue colour in the CIELAB space. We found that red and blue hues are on average not separated by 180°, and therefore that they do not seem to maximise hue disparity and thus conspicuousness amongst each other (see results).

Sumner and Mollon [32] previously suggested that the foliage background colouration has played an important role in the evolution of visual communication because the visual sensitivity of Old World primates is optimised for detecting targets against it. We therefore tested whether hue disparity could be influenced by the foliage background colouration. We studied two scenarios, postulating either that the red signal evolved first and the blue signal evolved secondarily to maximize the conspicuousness given the pre-existing red signals and the background colouration, or alternatively that the blue signal evolved first. To test the first scenario, we estimated which blue hue contrasted maximally against both the background colour and the red hue using a procedure of hue vectorisation. For background colouration, we used a dataset of approx. 5000 reflectance spectra of leaves recorded in tropical rain forests from Madagascar and Kenya (unpublished data). a* and b* coordinates were calculated as above for upper and lower leaf faces of each of the 144 plant species. Leaves from the two locations did not differ in hue (Wilcoxon test: *W* = 4902; *p* = 0.38) and were therefore pooled into a single data set. Vectorisation was made in CIELAB space by calculating 95% confident intervals (CI) of the coordinates of red faces and green leaf colours. Then, the bisector between red upper CI limit and green upper CI limit was drawn and its maximally contrasting hue was taken by adding 180°. The same was done with the lower CI limits. The angle between the limits of maximally contrasting hues indicates the range of hues maximising hue disparity against both the red face and the leaf colours. To test the second evolutionary scenario, we repeated the analyses taking the coordinates of blue faces and green leaves as fixed values to predict the theoretical red hue with maximal contrast. We used CI limits instead of calculating the theoretical maximally contrasting hue for each observed facial hue because variation in oxygen level can produce intra-individual variation in red hues [33].

### Signal information

#### Dominance rank

Following previous studies in mandrills [23], [34], we studied the role of facial colouration in signalling dominance rank. In male mandrills, dominance rank is positively correlated with offspring production and is thus related to males' fitness [35]. Male's dominance hierarchy has been demonstrated to be highly linear in this colony [34]. We updated male's dominance rank using *ad libitum* observations of the outcome of agonistic and approach-avoidance interactions during three weeks in March 2010. While the dominant positions were unequivocally assigned, the most subordinate males are often solitary and peripheral to the group and therefore do not encounter each other frequently [36]. For such males (35% of studied individuals), we assigned the same rank at the bottom of the social hierarchy. A low resolution of the most subordinate males occurred particularly in group E2 which contained 24 males. Twelve males were unambiguously ranked and twelve others were all assigned the same last rank. We used both absolute and relative ranks in the analyses. Both methods yielded similar results, only those obtained with absolute ranks are presented here.

#### Regression analyses

We performed General Linear Models (GLM procedure, SAS version 9.2) to study the relationships between colour attributes (*H* and *C* for red and blue and *CC* between blue and red) and dominance rank. We also included two other variables in the model: age and enclosure membership. First, secondary sexual traits change with age in male mandrills: males start to develop secondary sexual adornments, including facial colouration, around 6-yr old; the rate of changes in these adornments slows down after the age of 9-yr old [25]. Second, the 34 studied males live in three different enclosures that experienced different social conditions, notably due to different numbers of mature males. We expect male-male competition to be more intense in E2 (many mature males) compared to E1 or E3 (few mature males), with potential effects on colouration. In addition, the quality of rank resolution was different between E1 and E3 *vs.* E2, as mentioned above. We therefore considered the enclosure membership as a qualitative co-variate. Finally, we also included in our model the two interaction terms: enclosure*rank and age*rank. Our initial model included colour as dependent variable, rank, age, enclosure and interaction terms as independent variables. One outlier was removed from the analysis of red hue because this male presented an aberrant pattern (with blue reflection on the red patch). We used a Gaussian error structure for all models because the residuals were normally distributed (Kolmogorov-Smirnov test, results not shown). A backward model selection procedure was applied to select a best-fit set of explanatory variables, setting the threshold for significance to 0.05. We also ran a forward model selection yielding similar results.

#### Holistic perception of information accuracy

We compared the effect of perceiving colour patches individually or combined in a whole display on the accuracy with which information is perceived. We found that saturation but not hue attributes were correlated with male's rank (see results). We thus calculated the difference between all pairs of individuals in three colour measurements: blue saturation, red saturation and blue-red contrast in the perceptual space. For each colour measurement, the mean of the differences between all pairs was used as an estimate of information accuracy. We used Wilcoxon signed-rank tests to compare the different estimates of information accuracy with each other. Variances of differences between pairs were further compared using a non-parametric Fligner-Kileen test. High mean and variance values indicate improved perceptual abilities to discriminate between facial colourations of two males. Finally, we studied whether perceiving blue-red contrasts also increases discrimination of dominance categories. We split individuals into three groups based on rank. The first group only includes alpha males, because of the very large reproductive skew towards these males. The second group includes last-ranked peripheral males. The third group includes all other males. We used a Kruskal-Wallis test to compare means of blue-red contrast residuals (i.e. removing the effect of other predictors) between the three groups. The *p-value* of this test was then compared to *p-values* obtained when comparing means of blue saturation residuals and red saturation residuals.

### Information accuracy during the evolution of signal conspicuousness

Our results suggest that the evolution of red hue is constrained and that blue hue has evolved to increase the overall conspicuousness of the multicoloured face (see discussion). We then used a toy model consisting of two hypothetical males with different rank to study how selection on blue hue to increase the overall conspicuousness of the display influences information accuracy. Information accuracy estimated through the difference in blue-red contrast between the two males was thus compared with information accuracy estimated with difference in blue or in red saturation only, for different values of overall conspicuousness. Variation in the overall conspicuousness was generated by varying the hue of the ‘blue’ patch only. The ‘blue’ patch was allowed to take any hue present in the colour gamut of conspecifics (for simplicity we continued to call this patch the ‘blue’ patch independently on its hue). The hue of the ‘blue’ patch is defined by the angle separating it to the hue of the red patch: 0° and 360° describe a ‘blue’ patch with red hue, 180° describes the colour maximizing hue disparity and thus overall conspicuousness. We assumed that red hue is constant, that both males have the same ‘blue’ and red hues, and that the saturation of both colours is constant (set to 4 and 6 for the lower-ranked male and to 6 and 9 for the higher-ranked male, for the red and blue patches respectively).

Last, we repeated the analysis but considering the effect of the environment on the perception of the whole display. Here, blue-red contrast is replaced the mean between blue-red and blue-background contrasts. Note that this estimate of information accuracy cannot be quantitatively compared to previous estimates because averaging two contrasts has no physiological basis: it is a mathematical formulation that penalizes hues that are close to the background hue.

## Results

**Figure 1:**
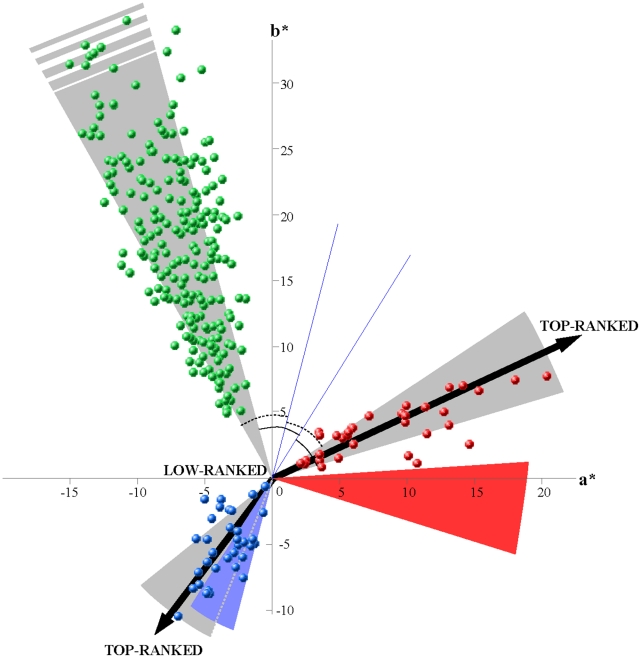
Loci of colour stimuli in the colour diagram reconstructed with the CIELAB model of colour vision. Green points represent the colours of leaves, blue and red points represent blue and red facial colours, respectively, in the a*b* plane of CIELAB. Leaves with outlying a*b* values are not represented (min a* = −18.4; max b* = 56.1). In CIELAB, one unit corresponds to one just noticeable colour difference. Grey shadings indicate 95% confidence interval of hues. Coloured shadings indicate predicted hues of maximal disparity against the other facial colour and the background. For example, the blue shading indicates the predicted blue hues of maximal contrast against both leaves and red colours. The construction of the blue shading is illustrated with blue lines and marks of equal angles. Top-ranked males are the most saturated for both blue and red colours as indicated by the arrows.

Red and blue hues are not separated by 180° in the CIELAB plane (Fig. 1). Red and blue hues therefore do not maximise hue disparity and thus conspicuousness to each other. When including foliage background colouration in the vectorisation procedure, however, half of the male mandrill faces show blue hues matching with the predicted range of hues that concomitantly maximise hue disparity against both the red facial hues and the foliage background. Conversely, there is no overlap between predicted and observed red hues (Fig. 1). Using a RNL model of colour vision instead of CIELAB yielded similar results (see Text S1).

We found that a male's dominance rank affects both red and blue colouration (Fig. 1). Higher-ranking males have more saturated blues and reds than lower-ranking males, and show consequently a stronger blue-red contrast (Table 1). Rank influences blue saturation and blue-red contrast differently in the enclosures: the relationships are stronger in E1/E3 than in E2. The influence of enclosure is likely due to the low resolution of the lower dominance ranks in E2 compared to E1 and E3. Finally, older males show more saturated red hues and consequently stronger blue-red contrast than younger males (Table 1). Using a RNL model of colour vision, we found similar relationships between dominance rank and colour attributes (see Text S1).

**Table 1:** Results obtained from the regression analyses.

Dependent var.	Predictors	*F*_1,32_ *	*P*
Blue saturation	age	0	0.99
	enclosure	3.62	0.07
	**rank**	**9.61**	**0.01**
	rank*age	1.04	0.32
	**rank** * **enclosure**	**6.31**	**0.02**
Blue hue	age	4.15	0.05
	enclosure	1.87	0.18
	rank	0.78	0.38
	rank*age	2.3	0.14
	rank*enclosure	0.06	0.81
Red saturation	**age**	**120.83**	**<0.0001**
	enclosure	3.08	0.09
	**rank**	**7.03**	**0.01**
	rank*age	1.58	0.22
	rank*enclosure	0.66	0.42
Red hue	age	1.26	0.27
	enclosure	4.16	0.05
	rank	1.89	0.18
	rank*age	0.03	0.86
	rank*enclosure	0.06	0.81
Blue-red contrast	**age**	**50.68**	**<0.0001**
	enclosure	1.04	0.32
	**rank**	**15.75**	**0.0005**
	rank*age	5.05	0.82
	**rank** * **enclosure**	**5.31**	**0.03**

*Except for Hr (df: 1, 31).

Significant relationships (*p<0.05*) are shown in bold.

Concerning the effect of holistic perception on the ability to discriminate two males based on their colour saturation, both the mean and the variance of differences between pairs of individuals in blue-red contrasts (*m* = 6.78/*σ* = 34.38) are significantly higher than means and variances of differences in blue (Wilcoxon signed-rank test: *m* = 2.84; *V* = 486110; *p<0.001*; Fligner-Kileen test: *σ* = 4.62; med χ^2^ = 764.61; *p<0.001*) and red saturation (*m* = 5.79; *V* = 368049; *p<0.001*; *σ* = 18.01; med χ^2^ = 831.05; *p<0.001*). These results indicate that it would be easier for a receiver to perceive differences in facial colouration between two males when evaluating blue-red contrast instead of evaluating only blue or red saturation. In addition, mean differences between the three rank groups were highly significant for blue-red contrast residuals (Kruskal-Wallis χ^2^ = 12.5, *p* = 0.0018) while being not significant for blue saturation residuals (KW χ^2^ = 5.68, *p* = 0.058) and for red saturation residuals (Kruskal-Wallis χ^2^ = 5.45, *p* = 0.065), although both are close to significance. Consequently, higher-ranked and lower-ranked males appear respectively more and less conspicuous than the population average when assessing the blue-red contrasts instead of variation solely within blue or within red.

**Figure 2:**
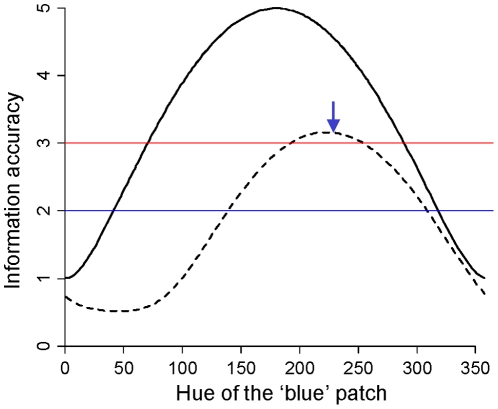
Relationship between information accuracy and the hue of the ‘blue’ patch. The hue of the ‘blue’ patch is given by the angle separating it to the fixed red hue. Information accuracy is either associated to the saturation of each colour perceived individually (blue and red horizontal lines), to the intra-signal colour contrast (continuous black line), or to the mean between the intra-signal colour contrast and the contrast between the colour with varying hue and the foliage background colouration. The range of hues where the black continuous line is above both the red and the blue lines describes bicoloured displays for which information is more accurately assessed from the perception of the ‘blue’-red contrast than from the perception of ‘blue’ or red saturation. The blue arrow indicates the mean blue hue effectively observed in the mandrill face.

Simulating various hues for the ‘blue’ patch revealed that the information accuracy estimated through the difference in red and ‘blue’ saturation between two males does not vary with overall conspicuousness (Fig. 2). Information accuracy is evidently constant because the saturation of colours is invariable by construction of the model. Using the difference in ‘blue’-red contrast, information accuracy varies with the overall conspicuousness: it is maximal when hue disparity is maximal (180°). Importantly, information accuracy so estimated is improved compared to information accuracy estimated though ‘blue’ or red saturation, but for high values of hue disparity only (the range of hues where the black continuous line is above both the red and the blue lines in Figure 2). Last, adding a penalty to the ‘blue’-red contrast when the ‘blue’ hue is close to the background hue revealed that information is most accurately perceived when the ‘blue’ hue is separated to the red hue by 230°. This theoretical ‘blue’ hue is close to the mean observed blue hue (Fig. 2).

## Discussion

Blue hues in mandrills are located in the perceptual space of primate colour vision so that they approximately maximise hue disparity against both the red hue and the foliage background colouration. In accordance with previous studies in mandrills (e.g. [23], ) and in the closely related drill *Mandrillus leucophaeus* [37], we found a positive correlation between dominance rank and the intensity of red colouration, even when the effect of age is taken into account. Moreover, we showed for the first time that dominant males exhibit more saturated blue colours than others and that the contrasts between facial blue and red increased conspecifics' abilities to discriminate social rank. Specifically, blue-red contrasts result in higher variance between males compared to the variance of males within each colour patch. Perceiving blue and red facial colours together can therefore improve the ability to discriminate both colour and rank among males, and thus information accuracy. Altogether, these results support that the evolution of red and blue colours are not independent and that the multicoloured face of male mandrills is a multicomponent signal.

### Evolution of the overall conspicuousness

In order to optimise the conspicuousness of a multicomponent signal, individual components should evolve to maximise the disparity among already existing hues. Half of the observed blue hues show maximal hue disparity against the combined vector of red hues and background colouration while there is no overlap between predicted and observed red hues. This result strongly suggests that blue hues but not red hues are a derived colour that evolved to maximally contrast against both a pre-existing facial colour and the dominant green environmental colouration. Red colouration is frequent in Old World primates and apparently spread in this group soon after the evolution of trichromacy [38]. In contrast, blue is a rare colour in mammals that has appeared only a few times independently [27]. It is thus likely that the red patches evolved first in mandrills, and that the chromatically very distant blue colouration is an innovation – i.e., a new colour differing in hue from pre-existing ones [10] – that evolved secondarily in a way that maximises the overall conspicuousness.

The finding that in mandrills the blue colour signals could have been selected to increase the conspicuousness of the complex display is in line with previous results on multicomponent signals [12], [17]. A strong disparity of colours in a complex display increases the overall conspicuousness of signals because it increases the stimulation of the sensory system of perceivers [10]. Because high stimulation of the sensory system is often associated with enhanced preference for the signaller (e.g., [39]), the evolution of conspicuousness through innovations therefore follows the model of sensory exploitation. This classical model for the evolution of epigamic traits predicts that sexually-selected traits evolve to exploit pre-existing preferences that occur in the sensory systems of perceivers [40]. We thus propose that sensory exploitation can explain the evolution of the multicoloured face of mandrills.

A striking result of our study is that the foliage background colouration could have influenced the evolution of the blue facial colouration in mandrills. The model of sensory exploitation predicts that the pre-existing sensory preferences are shaped by stimuli from the environment. In accordance with this prediction, Sumner and Mollon [32] previously showed that the colouration of leaves influenced the evolution of the visual system of Old World primates. The foliage background colouration should therefore play an important role in the evolution of social signals that are selected to be conspicuous in these primates. Although the background colouration is already known to influence the evolution of animal colouration [41], this result is, to our knowledge, the first evidence that background colouration in concert with pre-existing signaling colours can also influence the evolution of a derived colour patch in a muticomponent signal.

The pattern of hue disparity observed in mandrills differs from an optimal one in which both red and blues hues would maximally differ from leaf hues (i.e. blue, red and green hues are all separated by 120° in the a*b* plane of CIELAB). Differences in the mechanisms underlying the production of blue and red colours likely explain why the maximisation of hue disparity occurred in blue hues only. In mammals and birds, red skin colouration is induced by haemoglobins in blood flow [42]. The evolution of blood-mediated red signals is expected to be tightly linked to the physiological parameters of the signaller and therefore to be informative for the receivers. The hue of red signals is, however, strongly constrained by the vital and stable functions of haemoglobins. For example, the level of blood oxygenation causes variation in red hue [33]. Variation in red hue is therefore likely constrained in the context of signalling, which would explain why hue is not correlated with dominance or reproductive success in mandrills as in other primate species (see [19], [37]). In mandrill, blue colouration results from coherent scattering of light by quasi-ordered arrays of dermal collagen fibrils [27]. Although the evolution of blue hue may be constrained by the primary role of collagen to confer skin resistance, its variation is less tightly linked to vital functions, which is expected to relax constraints on its evolution. Based on our results in mandrills, we predict more generally that an increase in overall conspicuousness likely occurs with the stepwise development of innovations, but that an elaboration – i.e. a gradual selective process of design evolution [10] – could secondarily increase signal conspicuousness by adjusting the hue of those signal components that are less strongly linked to underlying physiological, developmental or behavioural traits, to the hue of pre-existing signal components.

### Relationship between overall conspicuousness and information accuracy

We showed that evaluating the blue-red contrast could allow a more accurate assessment of dominance than evaluating the saturation of single colour patches. This result shows that a holistic perception of the facial colouration of male mandrills has the potential to improve the accuracy of the information conveyed by each single component. Interestingly, the significant increase in information accuracy occurs because blue and red hues are strongly disparate. Our simulation of hue disparity indicates that the blue-red contrast would be less efficient in indicating dominance rank than red saturation if the ‘blue’ patch was not blue but, for example, orange (hue disparity between red and orange is approx. 50°). This result suggests that selection upon increased conspicuousness of the overall facial display could have simultaneously improved the information accuracy. In this way, the interactive evolution of conspicuousness and information accuracy would not be mediated by antagonistic trade-offs between these two signal features in mandrills, as it has been suggested in birds [17]. Instead, conspicuousness and information accuracy could evolve in synergy as a direct consequence of the intrinsic structure of the perceptual space of colour vision.

Identifying a pure receptor-based mechanism that can improve the assessment of dominance has important consequences for the evolution of multicomponent signals. Increased information accuracy resulting from the holistic perception of multicomponent signals has been, to our knowledge, invariably interpreted as a brain mechanism (both in primates and in other taxa such as fruitflies; [1], [43], [44]). Both the CIELAB and the RNL models of perceptual space used in this study have been developed for the perception of single colours. These perceptual spaces cannot therefore model signal properties that would emerge from the processing of multiple signal components by the brain. In addition, the RNL model is based on data from the peripheral visual system only. Our results thus show that although brain mechanisms can be involved, the peripheral sensory system could also –and possibly even alone– play an important role in increasing the accuracy of information associated with multicomponent displays.

### Hypotheses for the evolution of the mandrill multicoloured face

Because simple signals often contain errors in their information encoding [45], a strong selection to reduce errors could have favoured the evolution of two or more signals backing each other up [46]. Social systems exhibiting high variance in reproductive success show the strongest intensity of sexual selection [47]. In mandrills, reproductive skew among males is intense as dominant males sire more than 70% of offspring each year [35]. In addition, mandrills can live in large social groups of up to 800 individuals with many males moving in and out of the group [48]. Consequently, females should need reliable and precise estimates of male quality to choose among numerous potential mates, most of them likely being unfamiliar [49]. Similarly, relying on multiple informative traits could allow competitors to more reliably evaluate the ability of unfamiliar opponents to fight, and thus to avoid escalated fights [49]. Strong inter- and intra-sexual selection could thus have promoted the evolution of the multicoloured face in male mandrills.

The mechanisms of colour production could also have facilitated the evolution of multicoloured face. In mandrills, top-ranked males have the highest levels of circulating testosterone [36], which causes red saturated skins by peripheral vasodilatation after aromatization to estrogens [42], and certainly fur loss on the red patch via local testosterone receptors [50]. Blue colouration is caused by a locally special layout of the ubiquitous dermal collagen, with higher values of colour saturation resulting from thicker collagen layers [27]. Interestingly, collagen thickness is controlled by circulating androgens and the number of local androgen receptors [51], [52]. Thus both colours could be produced via a similar proximate mechanism, which may explain why both indicate male rank. From a proximal perspective, collagen accumulation and the resulting blue colouration could thus have arisen through the pre-existing physiological setting that underlies the red signalling system.

### Perceptual space as a tool to study the design of multicomponent signals

The perceptual space has become important in recent studies in communication because it allows studying signals in the eye of the beholder [53]. In this study, we further stress that the perceptual space is a suitable tool to analyse multicomponent signals. A key analysis here was the vectorisation of hues to predict a derived hue given two or more pre-existing hues. The vectorisation of hues should be of particular interest to study evolution of multicolour signals throughout phylogeny where the sequence of events can be predicted.

In this study, we used the CIELAB model of colour vision developed in humans. The geometry of the CIELAB space makes it particularly suited to study multicolour signals in catarrhine primates. Indeed, the CIELAB space is a modelled perceptual space originally reconstructed from estimated data on the peripheral visual system that has been subsequently transformed to match colour ordering systems reconstructed empirically (e.g., the Munsell colour system). Owing to this transformation, the CIELAB space is uniform (i.e. variations in hue and saturation are scaled uniformly across the entire space) meaning that CIELAB can be used to study colour differences at supra-threshold levels [30]. We thus suggest that the CIELAB model is well suited to study the evolution of multicolour signals made of strongly different hues in humans and in other catarrhine primates.

CIELAB has been developed in humans and its applicability to other primates depends on the similarities in the visual abilities between humans and non-human trichromatic primates. In general, the visual system of humans is very similar to that of other primates [54]. There are however small differences which mainly concern the sensitivity and the relative abundance of the photoreceptors sensitive to short wavelengths [54]. Such differences have led some authors working on non-human primates (e.g., [19]) to use another model that can incorporate species-specific parameters, namely the RNL model of perceptual space [31]. A major limitation of the RNL model is, however, that it was originally designed to study colour detectability. Like any perceptual space directly extracted from a photoreceptor excitation space, the RNL-derived space is not uniform and its validity at supra-threshold levels is still unknown [55]. Without further study, it is eventually conjectural whether CIELAB – which is a uniform perceptual space but which is based on human parameters – or RNL – which is not uniform but that can integrate species-specific parameters – best models colour vision in non-human primates. As a preliminary answer, we found that both models yielded similar results, thereby evidencing that CIELAB can be confidently used with Old World primates and that our vectorisation approach, which is fundamentally an Euclidean method, can be used with a non-uniform space (see also [12]). The fact that the RNL model accounts for the opponent nature of signals encoded by the retina likely explains why the RNL space is close to uniformity and thus that Euclidean geometry works within it. We thus suggest that both CIELAB and RNL as modelled perceptual spaces of colour vision offer a promising framework to study the evolution of multicolour signals in catarrhine primates.

Our suggestion that opponent models generate Euclidean-like space questions whether other perceptual spaces with similar properties could be used to study the evolution of multicoloured signals. The Luther diagram [56], a chromaticity diagram based on photoreceptor excitation and originally developed for humans, has already been adapted to non-human primates with known photoreceptor sensitivities in previous studies (e.g., [29], [57]). Like the RNL model, the Luther diagram is an opponent model of colour vision. Contrary to the RNL model, constructing a Luther diagram does not require any information relative to detection thresholds. The Luther diagram is thus less restrictive than the RNL model and could apply to a large array of species with known opponent mechanisms. Compared to both CIELAB and RNL models, however, the Luther diagram suffers from that its scaling is arbitrarily defined and thus does not spontaneously inform on the ability of a perceiver to differentiate close colours [58]. We thus suggest that CIELAB and the Luther diagram should be preferred when investigating colour discrimination among very distinct colours, but either CIELAB or the RNL model should be used in studies interested in discrimination among similar colours, or as in this study, in discrimination within and among colours.

## Supporting Information

Text S1 **Analysis of the mandrill multicoloured face with the Receptor Noise Limited model of colour vision.**
(DOC)
