# Supplementary material for: The Evolution of the Multicoloured Face of Mandrills: Insights from the Perceptual Space of Colour Vision
Source: PLoS One. 2011 Dec 21;6(12):e29117. doi: 10.1371/journal.pone.0029117 (PMC3244440; doi:10.1371/journal.pone.0029117)
Supplement: Text S1 — Analysis of the mandrill multicoloured face with the Receptor Noise Limited model of colour vision. (DOC) [file pone.0029117.s001.doc]

**Supporting Information**

**Text S1. Analysis of the mandrill multicoloured face with the Receptor Noise Limited model of colour vision.** In this appendix, we present calculations of colour attributes using the Receptor Noise Limited (RNL) model of colour vision [1]. We then use these new values of colour attributes for the analysis of hue disparity and the regression analyses (relationships between colour attributes and dominance rank). Results are presented in Figure S1 and in Table S1 at the end of this appendix. These results are qualitatively similar to those presented in the main text, which were gathered with the CIELAB model of colour vision.

*The RNL model of colour vision*

The RNL model of colour vision is presented in details in Vorobyev and Osorio [1] and in Kelber *et al.* [2]. However, because the original model has been variously modified in several studies, we detail here the equations we used to calculate values of colour attributes.

We first calculated the quantum catch *Q_i_* of a given photoreceptor type *i* as the integrated product over the wavelength λ of the receptor sensitivity *R_i_*(λ), reflectance spectrum *S*(λ), and illumination spectrum *I*(λ):

$$Q_{i}=\int_{\lambda} R_{i}(\lambda)S(\lambda)I(\lambda)d\lambda$$

We then apply the von Kries transformation to account of the phenomenon of colour constancy. The corrected quantum catch *f_i_* is therefore calculated as:

$$f_{i}=\ln\left(\frac{Q_{i-signal}}{Q_{i-Er}}\right)$$

where *Q_i–signal_* and *Q_i–Er_* are quantum catches corresponding to the signal (red or blue patches of the mandrill face, green leaves) and the environmental light, respectively.

Each photoreceptor type is assigned a noise factor *e_i_* based on a Weber fraction *ω* and the relative density *η_i_* of the photoreceptor type. In this study, we assumed photopic viewing condition, i.e. sufficiently bright illumination for the Weber law to hold [3]. The noise factor is therefore assumed to be independent on the perceived stimulus and is given by the neural noise only:

$$e_{i}=\frac{\omega}{\sqrt{\eta_{i}}}$$

Mandrills, like other catarrhine primates, use three types of photoreceptors for colour vision. The loci of colour signals can therefore be mapped into a two-dimensional chromaticity diagram by calculating colour coordinates as[4]:

$$\begin{matrix} X=A(f_{L}-f_{M}) \\ Y=B(f_{S}-(af_{L}+bf_{M})) \end{matrix}$$

where:

$$\begin{matrix} A=\sqrt{\frac{1}{e_{M}^{2}+e_{L}^{2}}} \\ B=\sqrt{\frac{e_{M}^{2}+e_{L}^{2}}{(e_{S}e_{M})^{2}+(e_{S}e_{L})^{2}+(e_{L}e_{M})^{2}}} \\ a=\frac{e_{M}^{2}}{e_{M}^{2}+e_{L}^{2}} \\ b=\frac{e_{L}^{2}}{e_{M}^{2}+e_{L}^{2}} \end{matrix}$$

Hue is defined as the direction of the colour vector in the chromaticity diagram and is given by the angular displacement (in radian) of the colour vector from the positive *X*-axis (see [5]). Saturation is defined as the distance between a colour loci and the achromatic origin of the plane. It is thus simply calculated as:

$$C=\sqrt{X^{2}+Y^{2}}$$

Note that *C* as calculated here is equivalent to ΔS as calculated by Vorobyev and Osorio [1]. Last, the blue-red contrast is defined as the Euclidean distance between the two loci in the diagram, i.e*.* as:

$$C_{blue-red}=\sqrt{(X_{blue}-X_{red})^{2}+(Y_{blue}-Y_{red})^{2}}$$

*Model parameters*

Receptor sensitivity *R_i_*(λ) were estimated with a rhodopsin template [6] fitted to the peaks of maximal spectral sensitivity (λ_max_). Because λ_max_ are not known for mandrill photoreceptors, we used the values found in *Macaca*, a genus closely related to the genus *Mandrillus* [7]. λ_max_ for macaque photoreceptors are 535 nm and 565 nm for M and L cones [8] and 430 nm for S cones [9]. Cone optical density at λ_max_ was assumed to be 0.3. The spectral optical density distribution of human lens and human macular pigment (retrieved from [10]) were used to correct sensitivities by the filtering of ocular media because these parameters are approximately constant among Old World primates [11,12]. Photoreceptor sensitivities were sampled every five nanometers. Following Endler and Mielke [13], integrals of photoreceptor sensitivities were normalized to 1.0. Each photoreceptor type therefore gives exactly equal output in white light, which is equivalent to have an eye adapted to white, grey or black backgrounds.

The illuminant *I*(λ) was the same as in the main text. In order to yield results that are comparable with those obtained using the CIELAB model, we defined *S*(λ) in *Q_i–Er_* as an achromatic (grey) stimulus.

In mandrills, S photoreceptors represent approximately 10% of the total cone population [14]. We assumed an equal proportion of M and L photoreceptors (as in macaques;[15]), so that the final relative density of photoreceptors *η_i_* is: 4.5(L):4.5(M):1(S). The noise *e_i_* for L and M photoreceptors was set to 0.02, a value close to human psychophysical thresholds [10]. We therefore evaluated *e_i_* for S photoreceptors as being approximately equal to 0.0425. This value is lower than in humans, which is due to the S photoreceptor population that is two to three times more prevalent in the mandrill (and macaque) retina than in the human retina [15].

**Figure S1 in Text S1.** Loci of colour stimuli in the colour diagram reconstructed with the RNL model of colour vision. Green points represent the colours of leaves, blue points and red points represent blue and red facial colours, respectively. Grey shading indicate 95% confidence interval of hues. Coloured shadings indicate predicted hues of maximal disparity against the other facial colour and the background. For example, the blue shading indicates the predicted blue hues of maximal contrast against both leaves and red colours. 22 out of 34 individuals have a facial blue hue that is within the predicted range of hues maximising the contract to red and green hues simultaneously.

**Table S1 in Text S1.** Results obtained from the regression analyses performed with perceptual attributes calculated using the RNL model of colour vision. Trends (*p<0.1)* and significant relationships (*p<0.05)* are shown in bold.

| **Dependent var.** | | | **Predictors** | | | ***F*_1,32_*** | ***P*** |
| --- | --- | --- | --- | --- | --- | --- | --- |
|  | Blue saturation | | age | | | 0.47 | 0.64 |
|  |  | | enclosure | | | 1.60 | 0.12 |
|  |  | | **rank** | | | **1.75** | **0.08** |
|  |  | | rank*age | | | 0.85 | 0.40 |
|  |  | | **rank*enclosure** | | | **1.71** | **0.09** |
|  | Blue hue | | age | | | 1.64 | 0.11 |
|  |  | | enclosure | | | 0.95 | 0.35 |
|  |  | | rank | | | 0.81 | 0.42 |
|  |  | | rank*age | | | 1.61 | 0.12 |
|  |  | | rank*enclosure | | | 0.30 | 0.76 |
|  | Red saturation | | **age** | | | **8.75** | **<0.0001** |
|  |  | | enclosure | | | 0.84 | 0.41 |
|  |  | | **rank** | | | **2.42** | **0.02** |
|  |  | | rank*age | | | 0.95 | 0.35 |
|  |  | | rank*enclosure | | | 0.09 | 0.92 |
|  | Red hue | | age | | | 1.13 | 0.26 |
|  |  | | enclosure | | | 0.16 | 0.87 |
|  |  | | rank | | | 1.23 | 0.22 |
|  |  | | rank*age | | | 0.27 | 0.79 |
|  |  | | rank*enclosure | | | 0.41 | 0.68 |
|  | Blue-red contrast | | **age** | | | **6.44** | **<0.0001** |
|  |  | | enclosure | | | 0.55 | 0.57 |
|  |  | | **rank** | | | **2.67** | **0.01** |
|  |  | | rank*age | | | 0.30 | 0.77 |
|  |  | | rank*enclosure | | | 0.92 | 0.37 |
| *Except for Hr (df: 1, 31) | | | | | | | |
|  | |  | |  |  | | |

**References**

1. Vorobyev M, Osorio D (1998) Receptor noise as a determinant of colour thresholds. P Roy Soc B-Biol Sci 265: 351-358.

2. Kelber A, Vorobyev M, Osorio D (2003) Animal colour vision - behavioural tests and physiological concepts. Biol Rev 78: 81-118.

3. Vorobyev M, Brandt R, Peitsch D, Laughlin SB, Menzel R (2001) Colour thresholds and receptor noise: Behaviour and physiology compared. Vis Res 41: 639-653.

4. Hempel DI, Giurfa M, Vorobyev MV (2001) Detection of coloured patterns by honeybees through chromatic and achromatic cues. J Comp Physiol A 187: 215-224.

5. Stoddard MC, Prum RO (2008) Evolution of avian plumage color in a tetrahedral color space: A phylogenetic analysis of new world buntings. Am Nat 171: 755-776.

6. Govardosvskii VI, Fyhrquist N, Reuter T, Kuzmin DG, Donner K (2000) In search of the visual pigment template. Vis Neurosci 17: 509-528.

7. Disotell TR (2000) Molecular systematics of the Cercopithecidae. In: Whitehead PF, Jolly CJ, editors. Old world monkeys. Cambridge: Cambridge University Press. pp. 29–56.

8. Bowmaker J, Mollon J, Jacobs G (1983) Microspectrophotometric results for old and new world primates. Col Vis Physiol Psychophys: 57-68.

9. Hárosi FI (1987) Cynomolgus and rhesus monkey visual pigments. Application of Fourier transform smoothing and statistical techniques to the determination of spectral parameters. J Gen Physiol 89: 717.

10. Wyszecki G, Stiles WS (1982) Color science: Concepts and methods, quantitative data and formulae. New-York: Wiley.

11. Cooper G, Robson J (1969) The yellow colour of the lens of man and other primates. J Physiol 203: 411.

12. Snodderly DM, Auran J, Delori F (1984) The macular pigment. II. Spatial distribution in primate retinas. Invest Ophthal Vis Sci 25: 674.

13. Endler JA, Mielke PW (2005) Comparing entire colour patterns as birds see them. Biol J Linn Soc 86: 405-431.

14. Ahnelt PK, Fernández E, Martinez O, Bolea JA, Kübber-Heiss A (2000) Irregular S-cone mosaics in felid retinas. Spatial interaction with axonless horizontal cells, revealed by cross correlation. J Opt Soc Am A 17: 580-588.

15. Calkins DJ (2001) Seeing with S cones. Prog Retin Eye Res 20: 255-287.
